# Identification and expression pattern analysis of the Hydroxycinnamoyl transferase gene family and function of StHCT6 in the potato

**DOI:** 10.3389/fpls.2026.1948389

**Published:** 2026-10-12

**Authors:** Chong Du, Xinyu Ni, Chengjian Sui, Xiaojie Luan, Xinyao Shi, Wenrui Zhao, Ziyi Zhang

**Affiliations:** Key Laboratory of Plants Adversity Adaptation and Genetic Improvement in Cold and Arid Regions of Inner Mongolia, College of Life Sciences, Inner Mongolia Agricultural University, Hohhot, China

**Keywords:** abiotic stress, gene expression, gene family, HCT, potato, VIGS

## Abstract

**Introduction:**

Hydroxycinnamoyl transferase (HCT) is a key enzyme in the plant phenylpropanoid biosynthetic pathway, involved in plant growth, development, and responses to biotic and abiotic stresses. As one of the world’s four major staple crops, potato (*Solanum tuberosum* L.) is widely cultivated due to its nutritional value and broad applications, highlighting the importance of studying the *StHCT* gene family and their expression patterns under abiotic stress.

**Methods:**

In this study, we identified 25 *HCT* genes from the Atlantic potato genome and conducted a comprehensive analysis of their phylogenetic relationships, gene structure, syntenic conservation across species, cis-regulatory elements, and expression specificity.

**Results:**

Tissue-specific expression profiles revealed higher transcript abundance in roots and stems compared to leaves and flowers. Notably, *StHCT* genes exhibited differential expression patterns under various stress conditions, with some genes consistently upregulated early during multiple treatments, suggesting their potential as core candidates mediating stress responses. Virus-induced gene silencing (VIGS) was used to silence *StHCT6*, which may have altered the disease resistance of potato leaves, resulting in more severe oxidative damage following bacterial infection.

**Discussion:**

Our results indicate that these *StHCTs* exhibit a high degree of conservation in terms of their genetic structure and evolutionary relationships. Some StHCTs show significant responses in tissue specificity and expression profiles under non-biological stress. After silencing *StHCT6* in potatoes, the leaves of the silenced plants suffered more severe oxidative damage after pathogen infection. These findings lay the foundation for elucidating the functional roles of *StHCT* in regulating potato growth and stress adaptation mechanisms.

## 1 Introduction

Potato (*Solanum tuberosum* L.), one of the world’s four staple crops, is rich in carbohydrates, protein, dietary fiber, vitamins, flavonoids and other active compounds and holds considerable promise for food development and industrial uses (Wang et al., 2023; Singh et al., 2021). Widespread climatic adaptation has allowed potato to be cultivated across the globe, yet the crop remains vulnerable to a range of abiotic stresses-such as nutrient deficiency, low and high temperatures, drought, and salinity-as well as biotic stresses from fungi, bacteria, viruses, and pests. Postharvest problems, like enzymatic browning triggered by mechanical damage, add further pressure (Tang et al., 2018; Qu et al., 2024; Handayani et al., 2019). All these factors can disrupt normal tuber growth and sharply reduce both yield and quality. Strengthening potato stress tolerance has therefore become a necessity for food security and industrial sustainability.

The phenylpropanoid pathway is among the most intensively studied metabolic routes in plants. During the growth process of plants, the phenylpropanoid metabolic pathway undergoes a series of reactions such as oxidation-reduction, hydroxylation, methylation, etc., which modify the side chains and benzene rings, thereby generating substances such as lignins, coumarin esters, phenolic acids, and flavonoids (Dixon and Barros, 2019). Chlorogenic acid, a phenolic acid, and various flavonoids can provide antioxidant protection by disrupting electron transport during reactive oxygen species(ROS) generation or by directly scavenging free radicals, helping to clear ROS that accumulate under environmental stress (He et al., 2020; Nakabayashi and Saito, 2015; Jiao et al., 2020). Lignin, a key component of secondary cell wall thickening, is deposited within the cell wall, where it supplies mechanical strength and structural support, facilitates long-distance water and nutrient transport, and forms a physical barrier against pathogen invasion-so protecting the plant while also playing an important role in stress responses (Zhao, 2016).

Hydroxycinnamoyl-CoA: shikimate hydroxycinnamoyl transferase (HCT), recognized relatively late in the study of lignin biosynthesis, acts as a key regulatory checkpoint at the C3 hydroxylation of the phenylpropanoid ring, governing the biosynthesis of guaiacyl (G) and syringyl (S) monolignols and directing the conversion of p-hydroxyphenyl (H) units into G/S lignin, thereby shaping lignin content and monomer composition (Wang et al., 2008; Hoffmann et al., 2003). Besides its role in lignification, HCT mediates the formation of chlorogenic acid from shikimate, caffeoyl-CoA and quinate within the phenylpropanoid pathway (Niggeweg et al., 2004; Alcázar Magaña et al., 2021). Researchers were the first to purify a hydroxycinnamoyl-CoA transferase from tobacco (*Nicotiana tabacum*) stems and identify it as the enzyme that, through a series of reactions, produces p-coumaroyl shikimate/quinate (Hoffmann et al., 2003). Studies have shown that strongly downregulating HCT expression in *Arabidopsis thaliana* disrupts auxin transport (Li et al., 2010). In both *Arabidopsis thaliana* and *Nicotiana benthamiana*, silencing *HCT* leads to dwarfism, alters lignin content and monomer composition, and-in *Arabidopsis*-shunts carbon flux away from lignification and into the flavonoid pathway, causing abundant flavonoid glycosides and acylated derivatives to accumulate while lignin synthesis is suppressed, ultimately driving a buildup of flavonoids (Hoffmann et al., 2004; Besseau et al., 2007).

Work by Shadle et al. and Gallego-Giraldo et al. demonstrated that suppressing *HCT* in alfalfa (*Medicago sativa*) not only stunts growth and delays flowering, but also reduces and remodels lignin content and composition. At the same time, levels of salicylic acid (SA), jasmonic acid (JA), and abscisic acid (ABA) rise, pathogenesis-related (PR) and stress-responsive genes are activated, and resistance to fungal pathogens and drought stress is markedly enhanced (Shadle et al., 2007; Gallego-Giraldo et al., 2011). This response has been attributed to the release of bioactive fragments and hydrogen peroxide from compromised cell walls, which together trigger a systemic defense response.Wagner et al. used RNA interference (RNAi) to silence *HCT* in callus of the gymnosperm radiata pine (*Pinus radiata*), which led to reduced lignin content in subsequently differentiated tracheary elements (Wagner et al., 2007). Silencing *HCT* in herbaceous peony (*Paeonia lactiflora*) similarly decreased the number of secondary cell wall layers in flowering stems and markedly suppressed lignin accumulation (Xu, 2022). Wang et al. showed that the maize (*Zea mays*) genes HCT1806 and HCT4918 participate in defense regulation and disease resistance responses (Wang et al., 2015). Lu found that overexpressing *HCT* in poplar (*Populus*) raises lignin levels, thickens cell walls, and produces tightly packed fibers, thereby deterring feeding by longhorn beetle larvae (*Cerambycidae*) (Lü, 2010). In tea (*Camellia sinensis*), *HCT* is upregulated upon infestation by the tea green leafhopper (*Empoasca vitis*), reinforcing secondary metabolite-based defense through the chlorogenic acid pathway (Sun et al., 2018). Notably, HCT function appears to be species-specific: silencing *HCT* in barley reduces lignin while enhancing defense responses (Nelson et al., 2017), whereas in *Arabidopsis* it is accompanied by growth defects, hinting that *HCT* may balance growth and defense through differential metabolic flux partitioning in different plants. These findings point to new strategies for improving crop stress tolerance via *HCT*. Collectively, these results underscore the multifunctional importance of *HCT* genes in coordinating developmental processes and tolerance to both abiotic and biotic stresses.

In this study, we systematically identified *StHCT* multiple genes from the potato genome (*Solanum tuberosum* L.) and characterized their structural features, phylogenetic relationships, and evolutionary divergence. Tissue-specific expression profiling revealed distinct transcript abundances of *StHCT* genes in roots, stems, leaves, and flowers. Using RNA-seq data and qRT-PCR, we analyzed *StHCT* expression patterns under various abiotic stresses (high/low temperature, NaCl, mannitol) and hormone treatments-including ABA, indole-3-acetic acid (IAA), and gibberellic acid (GA_3_)-and identified *StHCT6* as a promising candidate. Subcellular localization and gene silencing in potato were then carried out to preliminarily explore its function. These findings offer important insights into the functional specialization of *HCT* genes during potato development and provide a theoretical basis for further mechanistic investigation.

## 2 Materials and methods

### 2.1 Whole-genome identification and physicochemical property analysis of the *StHCTs* genes

The genome and annotation files, protein sequences, etc. of the potato ‘Atlantic’ were obtained from the potato database (spuddb.uga.edu). The HMM model of the conserved domain (PF02548) in the HCT protein was downloaded from PFam (https://www.ebi.ac.uk/interpro/entry/pfam/#table). The potato protein sequences were compared with the Arabidopsis HCT protein sequences in the NCBI database using BLASTP. The BLASTP threshold was set at E-value ≤ 1e-5, and the sequence coverage ≥ 50%, and the domain coverage ≥ 50%. The genes were annotated and searched through the eggNOG online website (http://eggnog-mapper.embl.de/), HMMER3.0 (http://hmmer.org/download.html), and NCBI-CDD (https://www.ncbi.nlm.nih.gov/Structure/bwrpsb/bwrpsb.cgi?22). Finally, based on the above results, the potato StHCTs gene family was analyzed and identified at the whole genome level. Genes were named sequentially according to their physical order on chromosomes chr01 through chr12-for instance, the locus Soltu.Atl_v3.03_1G017870.1 was designated *StHCT1*. The number of amino acids, molecular weight, isoelectric point, and grand average of hydropathicity (GRAVY) were predicted via ExPASy (https://www.expasy.org/), while subcellular localization was predicted with WoLF PSORT (https://wolfpsort.hgc.jp). Chromosomal start and end positions of all genes were obtained from the Potato Database, and a scaled physical map of the *StHCTs* loci across the 12 chromosomes was visualized using TBtools (Chen et al., 2020).

### 2.2 Gene replication and correlation analysis

Collinearity analysis of *StHCTs* was performed using MCScanX, and the ratio of non-synonymous to synonymous substitutions (Ka/Ks) for duplicated gene pairs was computed with KaKs_Calculator. Synteny relationships between potato *StHCTs* and those from other species-*Arabidopsis thaliana*, *Oryza sativa* (rice), *Solanum lycopersicum* (tomato), and *Solanum melongena* (eggplant)-were visualized via the Dual Synteny Plot function in TBtools. Genome sequences and annotation files for these species were retrieved from Ensembl Plants (https://plants.ensembl.org/index.html) and the Eggplant Genome Database (http://eggplant.kazusa.or.jp/index.html), respectively.

### 2.3 Genetic structure analysis

Gene structure annotation in GFF format was downloaded from the Potato Database, and the exon–intron organization of *StHCT* genes was delineated using TBtools. Conserved motifs in the StHCT proteins were identified with the online MEME suite (https://meme-suite.org), setting the maximum number of motifs to 10, and conserved amino acid domains were predicted through the NCBI Conserved Domain Database (CDD).

### 2.4 Phylogenetic analysis and multiple sequence alignment analysis

The HCT proteins of potato and *Arabidopsis thaliana* and other species were compared using MEGA 7 software (multiple species protein sequences were retrieved from NCBI). A phylogenetic tree was constructed using the Neighbor-joining method. The substitution model was Poisson model and the number of Bootstrap replications was 1000, with other parameters set to default. The phylogenetic tree was optimized using the online software Evolview v (https://www.evolgenius.info/evolview). Multiple sequence alignments were performed using DNAMAN software.

### 2.5 Analysis of cis-regulatory elements

The 2,000 bp sequences upstream of the start codons of these genes were obtained from the potato genome. The cis-elements in the promoter sequences of these genes were retrieved and annotated online through the Plant CARE database (https://bioinformatics.psb.ugent.be/webtools/plantcare/html).

### 2.6 Analysis of gene organization expression patterns and stress response expression patterns

Using the RNA-Seq data retrieved from the PGSC database (NCBI BioProject PRJNA753086), for each gene model, the transcript quantity was quantified using the Kallisto (v0.51.0) software in units of transcripts per million (TPM). The gene expression patterns of *StHCTs* genes under different tissues and different stress conditions were studied and analyzed. The processing details for the partial treatment are as follows: Leaf Salt Treatment: Mature leaves, plants approximately 1 month old, 100ml of 150mM Sodium Chloride Solution, applied to the pots 24 hours prior to sampling; Leaf Salt Control: mature leaf, Control for salt and drought leaf; Leaf MeJA Treatment: mature leaf, ~1 month old plant, 250uM MeJA solution in water/ethanol, spray ~5 times to drench leaves; Leaf MeJA Control: mature leaf, Control for methyl jasmonate leaf; Leaf Heat Treatment: mature leaf, ~1 month old plant, Watered before treatment to ensure no drought stress, 37°C Day/28°C Night for 24hrs; Leaf Heat Control: mature leaf, Control for heat leaf; Leaf Cold Treatment: mature leaf, ~1 month old plant, Cold treatment at 10°C for 24 hours; Leaf Cold Control: mature leaf, Control for cold leaf; Leaf BTH Treatment: mature leaf, ~1 month old plant, 100ug/ml active ingredient, spray ~5 times to drench leaves; Leaf BTH Control: mature leaf, Control for BTH leaf; Leaf Drought Treatment: mature leaf, ~1 month old plant,Plants had water withheld for 7 days, plants had wilted leaves exhibiting drought phenotype for collection; Root Salt Treatment: root, ~1 month old plant, 100ml of 150mM Sodium Chloride Solution to pots 24 hours prior to sampling; Root Drought Treatment: root, ~1 month old plant,Plants had water withheld for 7 days, plants had wilted leaves exhibiting drought phenotype for collection; Leaf 10pm: immature leaf 10pm harvest; Leaf 6pm: immature leaf 6pm harvest; Leaf 2pm: immature leaf 2pm harvest; Leaf 10am: immature leaf 10am harvest; Leaf 6am End: immature leaf 6am end harvest; Leaf 6am Start: immature leaf 6am start harvest; Leaf 2am: immature leaf 2am harvest; Hooked stolon: Terminal hooked stolon tip, harvested 0.5 cm of tip; Swollen stolon: Swelling stolon tip, sometimes flattened hook with a terminal end that is clearly enlarging but no roundness is present yet, harvested 2cm of tip; Root: Control for salt and drought root treatment.

### 2.7 Plant materials and stress treatments

The potato cultivar ‘Atlantic’ (*Solanum tuberosum* L.) used in this study was provided by the College of Life Sciences, Inner Mongolia Agricultural University, and plants were grown in a growth chamber (22°C, 60% humidity, 16/8 h light/dark photoperiod, 6 000 lx illumination) in their Plant Stress Molecular Laboratory. To characterize the expression patterns of *StHCT* genes under different abiotic stresses and exogenous hormone stimuli, uniform, vigorously growing 30-day-old potato plants were selected. Stress treatments were applied following Chang et al. and Shi et al: with normally grown plants as the control, the plants were exposed to high temperature (42°C), low temperature (4°C), salt (250 mM NaCl), and drought (20% PEG 6000), respectively. Using mock spraying with distilled water as the control, leaves were separately sprayed with indole-3-acetic acid (10 µM IAA), gibberellic acid (50 µM GA_3_), or abscisic acid (10 µM ABA) (Chang et al., 2024; Shi et al., 2025). After treatment, the plants were returned to the growth chamber; at 2, 4, 8, 12, and 24 h post-treatment, leaf samples were harvested, wrapped in aluminum foil, flash-frozen in liquid nitrogen, and stored at −80°C . Three biological replicates were taken for each sample.

### 2.8 Real-time fluorescence quantitative PCR (qRT-PCR)

The qTOWER^3^ real-time fluorescence quantitative PCR system of JENA Company was used to conduct qRT-PCR analysis of the *StHCTs* gene. The primer sequences (**Supplementary Table**) were designed using Primer-BLAST. Total RNA was extracted from each collected sample using the Total RNA Rapid Extraction Kit (ER501-01, TransGen Biotech, Beijing), and cDNA was synthesized using TransScript^®^ One-Step gDNA Removal and cDNA Synthesis SuperMix (AT311, TransGen Biotech, Beijing). The qRT-PCR reaction was performed using the ChamQ Universal SYBR qPCR Master Mix (Q711, Vazyme, Nanjing) reagent kit. *EF1α* was used as the internal reference gene. The amplification reaction system and reaction program can be found in the fluorescence quantitative PCR manual (Tang et al., 2017). The data from three independent repetitions were calculated using the 2^-ΔΔCt^ method (Bustin et al., 2009).

### 2.9 Interaction protein analysis

The interaction network of StHCT proteins was analyzed based on the interaction relationships in the STRING protein interaction database (http://string-db.org). The GO and KEGG enrichment information was directly extracted from STRING. The interaction relationship network diagram was constructed using Cytoscape.

### 2.10 Cloning of the CDS sequence of the *StHCT6* gene, construction of fusion expression vectors and transient expression

Take potato leaves, extract total RNA and check its integrity. Use the reverse transcription kit as the reverse transcription template for cDNA synthesis. Design primers using SnapGene software, then amplify the potato *StHCT6* gene sequence and clone it into the transient expression vector pCAMBIA2300-eGFP. The method of transient infiltration of tobacco is referred to Mo et al (Mo et al., 2023). Then, use the laser confocal microscope (37081, Carl Zeiss, Germany) to observe the localization of the *StHCT6*-eGFP fluorescence signal.

### 2.11 Construction of *StHCT6* silencing vector and plant infection

Primers were designed to amplify the optimal silencing site (VIGSStHCT6) (**Supplementary Table**) of the *StHCT6* gene from SGN-VIGS (https://vigs.solgenomics.net/) onto the pTRV2 vector. The plasmids pTRV1, pTRV2, pTRV2-*PDS* and pTRV2-*StHCT6* were co-transferred into Agrobacterium GV3101. The resuspended pTRV2, pTRV2-*PDS* and pTRV2-*StHCT6* were mixed with pTRV1 in equal volumes and left to stand at room temperature for 4 hours to obtain the TRV infection solution. VIGS transient infection was performed on 4-week-old potato leaves. During the injection process, the main veins of the potato leaves were avoided. After dark incubation for 2 days, the leaves were transferred to an artificial growth chamber for normal management. The light bleaching symptoms of the potato leaves after pTRV2-*PDS* infection were observed to confirm the successful infection. The *StHCT6* gene silencing plants were identified using the qPCR method.

### 2.12 Potato leaf bacterial inoculation treatment and disease resistance verification

For detached leaf assays, sterile filter paper was placed in Petri dishes and moistened with sterile water, and the petioles were wrapped with wet sterile cotton to keep them hydrated. Using a needleless syringe, 20 µL of Ralstonia solanacearum (RS) suspension (OD_600_ = 0.8) was infiltrated into one-quarter of the leaf surface of healthy, disease-free wild-type potato leaves, allowing the inoculum to spread along the veins. The dishes were sealed to maintain humidity and incubated at room temperature under a 16 h light photoperiod. Leaf symptoms were recorded 1, 2, 3, 4, and 5 days after inoculation, and lesion areas were quantified with ImageJ. Leaves were collected from silenced (VIGS) and wild-type (WT) potato plants at 0 and 5 days post-inoculation (dpi) with the pathogen. The activities of superoxide dismutase (SOD), peroxidase (POD), and catalase (CAT), as well as the contents of malondialdehyde (MDA) and lignin, were determined using commercial assay kits (Suzhou Geruisi Biotechnology Co., Ltd., Suzhou, China) according to the manufacturer’s instructions.

### 2.13 Statistical analysis

The number of biological and technical replicates for this experiment was all more than 3 times. The data results are presented as the mean ± standard deviation. Statistical analysis and significance tests were conducted using Microsoft Office Excel 2019, SPSS, GraphPad Prism 10, and Origin 2024.

## 3 Results

### 3.1 Genome-wide identification and physical-chemical property prediction of StHCTs genes in potatoes

In this study, 25 members of the *HCT* gene family were identified in potatoes, and they were renamed *StHCT1* to *StHCT25* based on their positions on the chromosomes. The physical and chemical information of these genes and their corresponding proteins, as well as the chromosome positions, are shown in **Figure 1A** (**Supplementary Table S1**). The lengths of StHCTs range from 433 aa (StHCT3/5) to 468 aa (StHCT9/13/18), with similar lengths. The molecular weight ranges from 47,964.33 Da (StHCT3) to 52,016.38 Da (StHCT18), the theoretical isoelectric point (pI) ranges from 5.22 (StHCT3) to 8.86 (StHCT14), the aliphatic index ranges from 79.91 (StHCT25) to 99.67 (StHCT19), the hydrophilicity value ranges from -0.349 (StHCT25) to 0.07 (StHCT22), and the hydrophilicity is close. The instability index of StHCTs ranges from 34.15 to 45.58 (> 40 indicates unstable protein, < 40 indicates stable protein). The subcellular prediction results show that the StHCTs genes are located in the cytoplasm (12), chloroplast (10), and peroxisome (3). The chromosome localization results of the StHCTs genes based on the chromosome annotation information of potatoes are shown in **Figure 1B**. The 25 *HCT* genes are distributed on multiple chromosomes, where chr7_1 and chr07_2 contain 4 and 5 *StHCTs* genes respectively, and the remaining listed chromosomes contain 1 to 2 StHCTs genes. The chromosome length is scaled in megabytes (Mb, left axis). The red marks indicate the physical position of *StHCT* members on the chromosome. The gradient of red, blue, and white shading represents the regional gene density, with darker tones corresponding to higher gene concentration.

**Figure 1:**
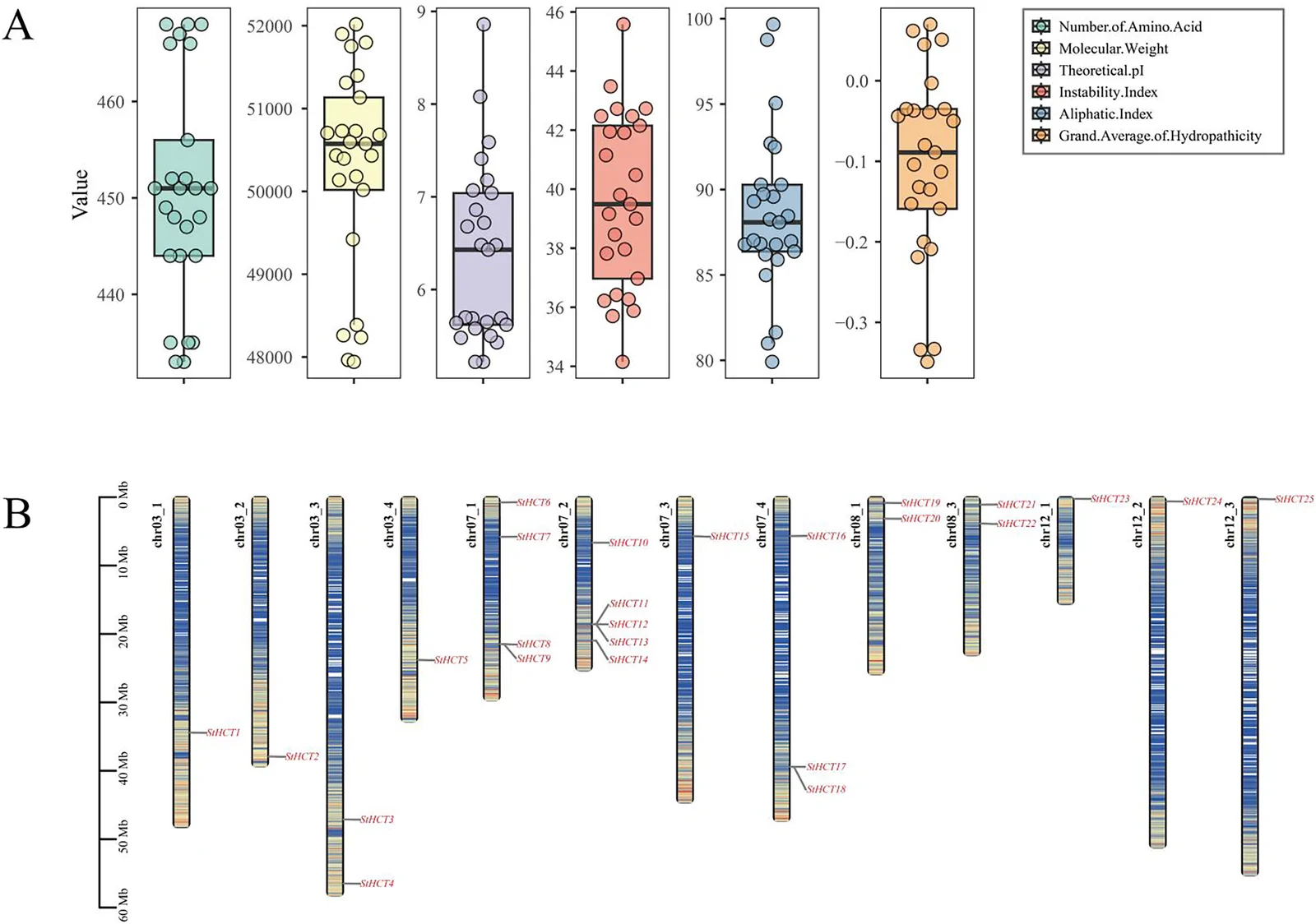
**(A, B)** The physical and chemical properties of the *StHCTs* gene and chromosomal localization of *StHCTs* genes on potato genome.

### 3.2 Repeated analysis of *StHCTs* genes

Fragment duplication and tandem duplication are key factors for the expansion of gene families. To study the duplication events of the StHCTs genes, a collinearity analysis was conducted on these identified genes, revealing that there were 12 *StHCTs* genes involved in 13 pairs of duplicated gene pairs (**Figure 2A**; **Supplementary Table S2**). Notably, *StHCT7*, *StHCT10*, *StHCT15*, and *StHCT16* each participated in 3 duplication events, while the other members participated in 1 to 2 duplication events. The non-synonymous substitution rate (Ka) and synonymous substitution rate (Ks) are the basis for evaluating whether the collinear genes have been subjected to selection pressure. Ka/Ks < 1: purifying selection, Ka/Ks = 1: neutral evolution, Ka/Ks > 1: positive selection. After conducting the selection pressure analysis in this study (**Figure 2B**), the evolutionary Ka/Ks ratios of the 13 duplicated gene pairs ranged from 0.0864 to 0.3403, all <1, indicating that these *StHCTs* genes from the same species underwent strong purifying selection after the duplication events, and their functions were relatively conserved. A low Ka/Ks ratio indicates that non-synonymous mutations are largely excluded. This phenomenon is typically observed in genes that are functionally important and conserved, such as those encoding key metabolic enzymes. In **Figure 2A**, the rings from the outside to the inside represent the ‘Atlantic’ tetraploid potato chromosome scaffold (different colors represent different chromosomes, the outer scale is in megabytes), gene density heat map (red indicates high gene density, yellow indicates low gene density), GC ratio histogram (the higher the GC ratio, the higher the histogram), GC skew point plot (the relative content of guanine and cytosine in the chromosome sequence is represented by yellow and red dots), and N ratio bar plot (the more unknown bases a chromosome contains at this position, the higher the red bar), and the collinear pairs of *StHCTs* genes are highlighted in red.

**Figure 2:**
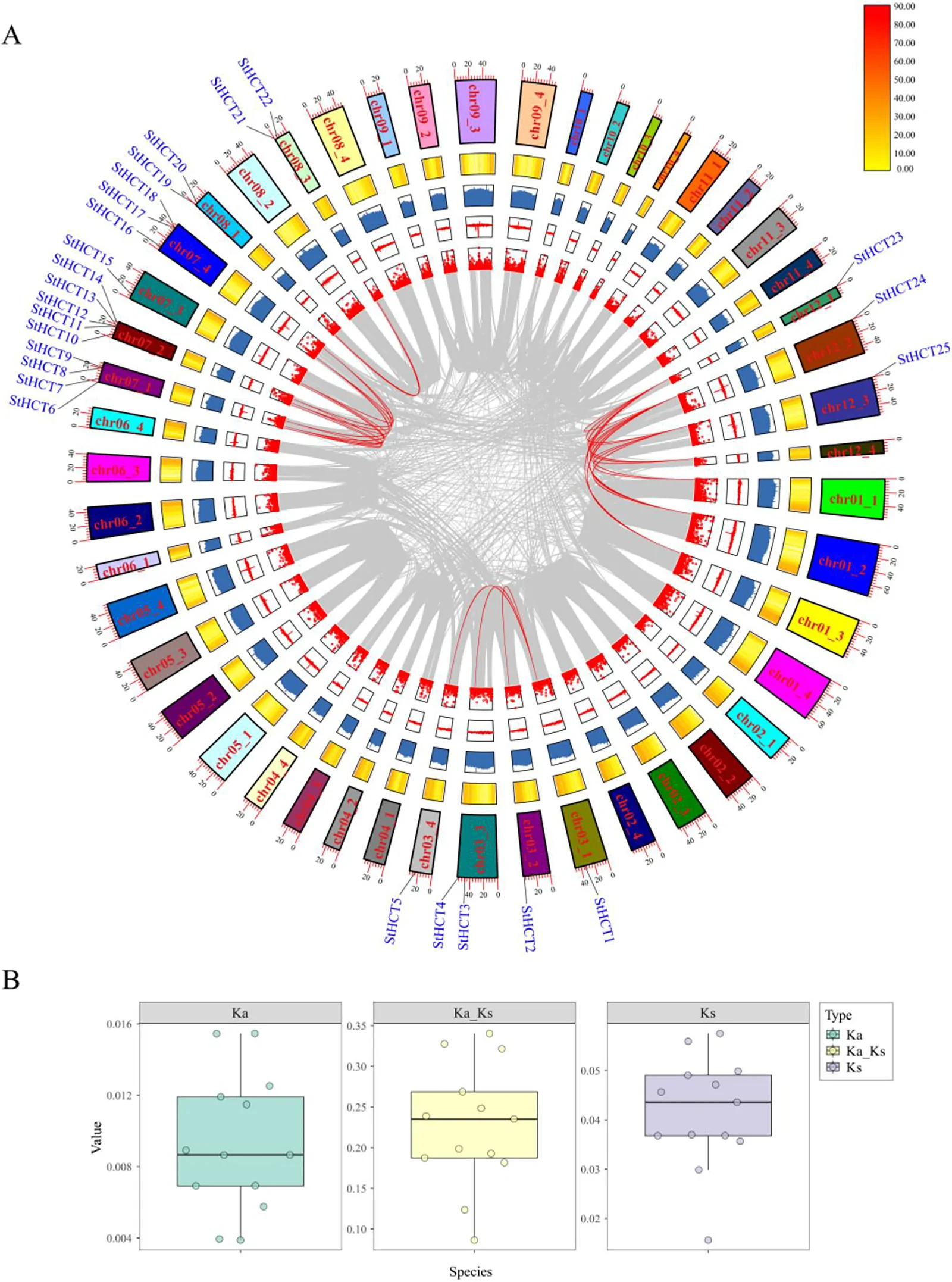
**(A, B)** Homology analysis of *StHCT* genes in potato genome and Ka/Ks values of gene co-linearity pairs.

### 3.3 Co-linearity analysis of *StHCTs* genes

To study how genes expand in the genome of a species and to reveal the similarities between *StHCTs* and other homologous genes of the same species, as well as their evolutionary relationships, a co-linearity analysis network diagram was constructed for species such as *Arabidopsis thaliana*, rice (*Oryza sativa*), tomato (*S. lycopersicum*), and eggplant (*S. melongena*) based on the gene sequences of potatoes (**Figure 3**; **Supplementary Table S3**). The results show that the co-linearity relationship between potatoes and eggplants, tomatoes is higher than that between *Arabidopsis thaliana* and rice, with the number of co-linearity genes being 14 and 7 respectively. The number of co-linearity genes between potatoes and solanaceous plants is mainly concentrated on chromosomes chr3, chr7, chr8, and chr12; the number of co-linearity genes with *Arabidopsis thaliana* and rice is 1, concentrated on chromosome chr3. The analysis indicates that these homologous gene pairs may have existed before the differentiation of the ancestral lineage, and the co-linearity retained genes are relatively conservative and may have core functions. They are stably present in the cross-species co-linearity blocks and also suggest that the ‘Atlantic’ *StHCTs* gene family of potatoes may have expanded and evolved through gene duplication. The co-linearity gene pairs between *HCT* genes of potatoes and those of *Arabidopsis thaliana*, rice, tomato, and eggplant are highlighted in red. The gray lines represent the gene blocks in potatoes that are homologous to other genomes.

**Figure 3:**
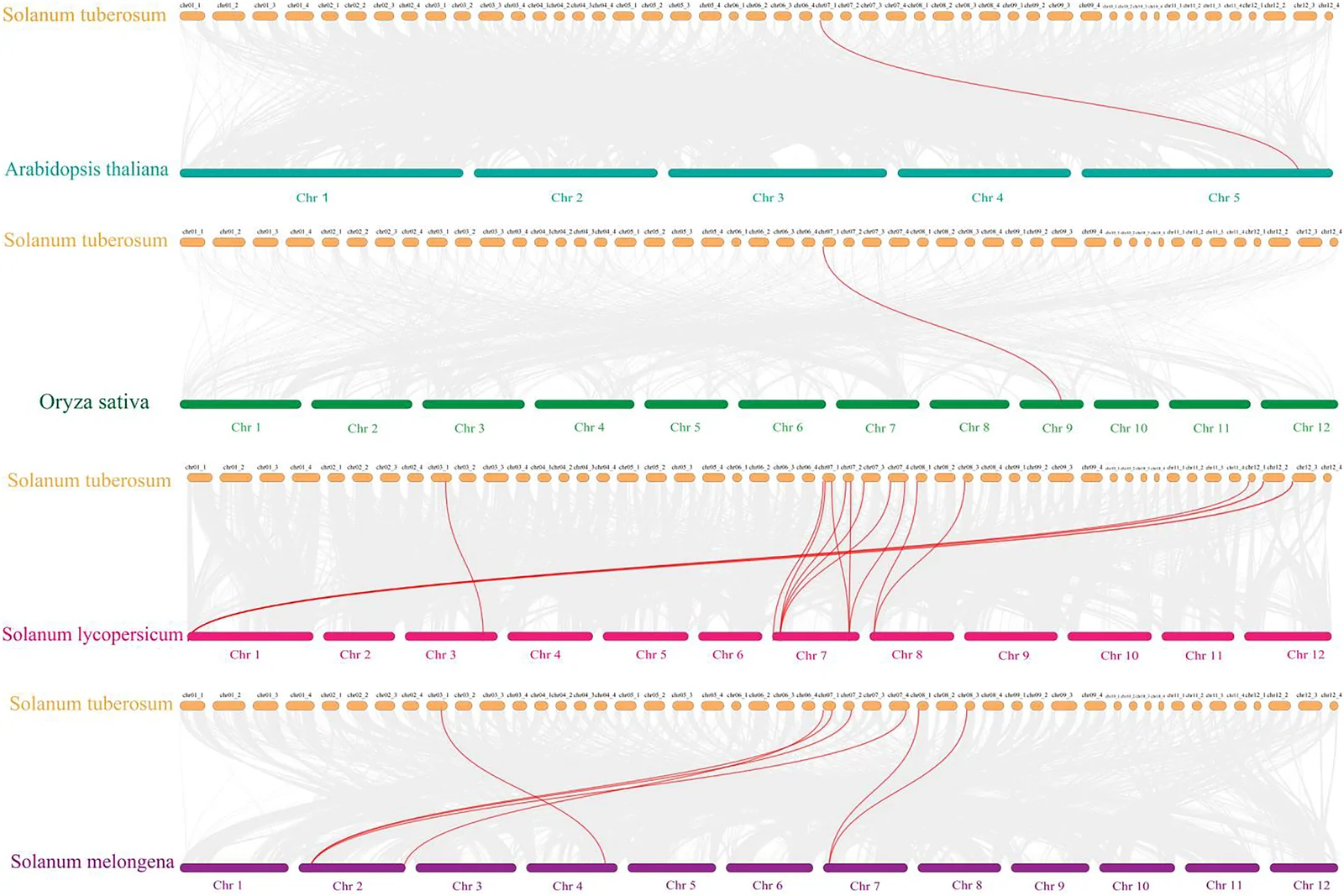
Analysis of the homology of the *HCT* gene among potatoes and four other plants.

### 3.4 Evolution, conserved motifs, conserved domains, and gene structure analysis of *StHCTs* genes

To investigate the evolutionary relationships among the individual members of the potato StHCTs genes, an adjacent (N-J) tree was constructed based on the nucleotide sequences (**Figure 4A**). The 25 StHCTs genes clustered into 6 subfamilies, with subfamilies I and VI containing more genes. The MEME online program was used to predict and analyze the conserved motifs of StHCTs proteins, and 10 different motifs were identified (**Figures 4B, E**; **Supplementary Table S4**). Motif 8 was only distributed in subfamilies I and II, while the other 9 motifs were present in all 25 StHCTs proteins. The composition and distribution of motifs were relatively conserved among the subfamilies, and the types, composition, quantity, and positions of motifs in the genes of the same subfamily were also relatively similar. Among them, motif 1 contains the HXXXD conserved sequence, and motif 4 contains the DFWGG conserved sequence. HXXXD is the catalytic recognition center of HCT proteins, and DFWGG can help maintain the stability of the protein. These two motifs are shared by the HCT gene family, and all 25 StHCTs genes contain these two motifs. This indicates that they are relatively conserved during evolution and are necessary for maintaining their own protein functions.

**Figure 4:**
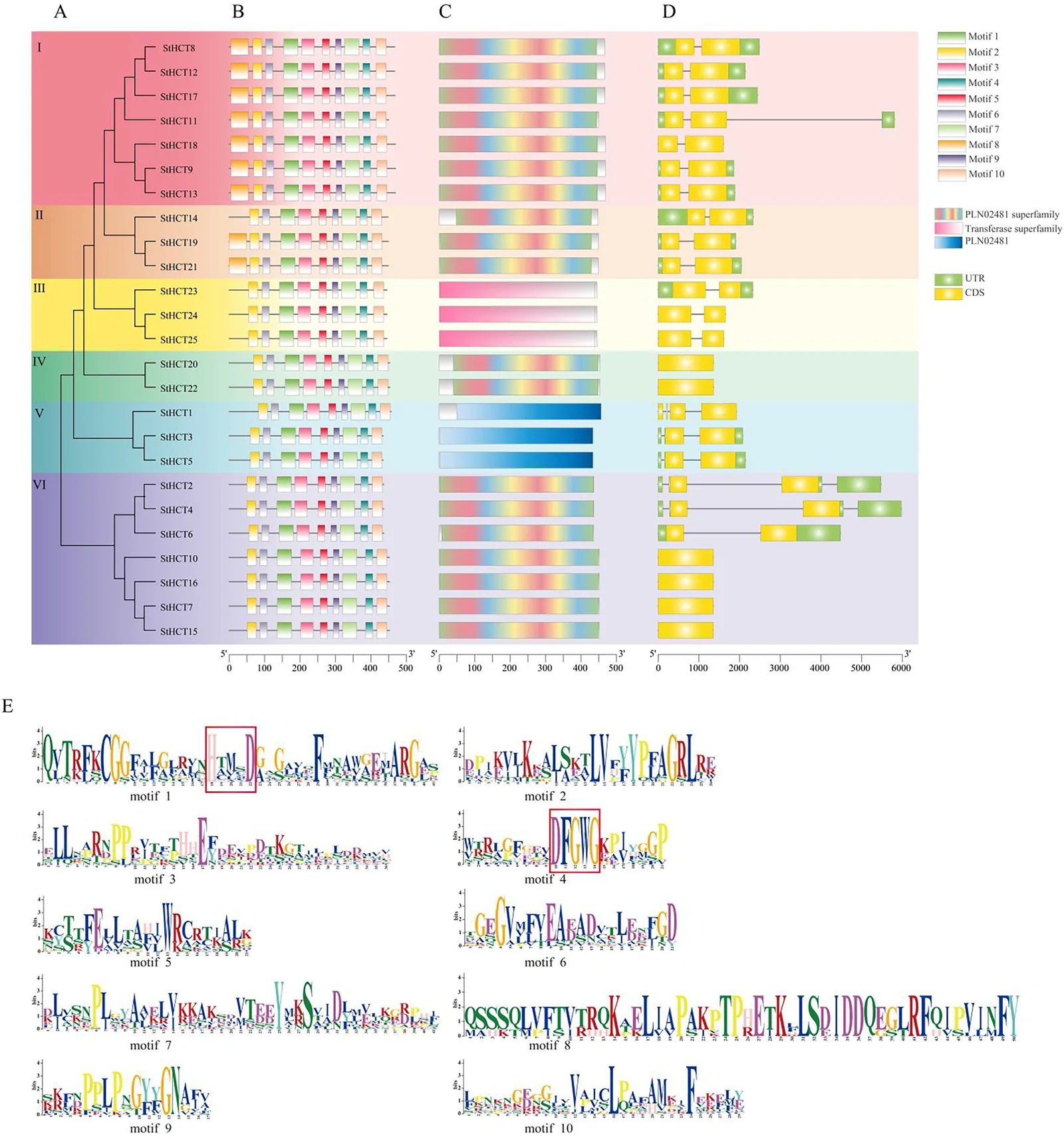
**(A–E)** Conserved motifs, conserved domains and gene structures of *StHCT* based on evolutionary relationships.

The conservation domains of StHCTs proteins were explored through NCBI CDD to determine their superfamily and family levels. The results are shown in **Figure 4C**. The amino acid sequences of 25 StHCTs proteins all contain conserved domains of the transferase superfamily and PLN02481 superfamily. The transferase superfamily is the core functional region of hydroxycinnamoyl transferase; the PLN02481 superfamily is closely related to the acyltransferase function in plant secondary metabolism. These conserved domains occupy most of the protein sequence and are highly conserved. Members of the same subfamily have similar domains, such as the domain structures of motifs in subfamily III belonging to the transferase superfamily, and the other subfamilies belonging to the PLN02481 superfamily.

In addition, the intron-exon distribution of *StHCTs* genes in the potato ‘Atlantic’ was analyzed by comparing the CDS sequences and complete gene sequences. The results are shown in **Figure 4D**. The number of exons and introns of the 25 *StHCTs* genes ranged from 0 to 3. Some genes had a simple single-exon structure and had no 5’UTR or 3’UTR (*StHCT7*, *StHCT10*, *StHCT15*, *StHCT16*, *StHCT20*, *StHCT22*); some other genes contained introns but lacked 5’UTR and 3’UTR (*StHCT18*, *StHCT24*, *StHCT25*). The coding regions of *StHCT2*, *StHCT4*, *StHCT6*, and *StHCT11* had a high degree of sequence homology, and this region was separated by a long intron. The sequence homology and intron-exon structure positions and quantities of members within the same subfamily were similar, which also indicates that these genes may have undergone gene duplication events during evolution and may have similar functions in certain specific physiological activities of potatoes in some subfamilies.

### 3.5 Phylogenetic analysis and amino acid sequence analysis of *StHCTs*

To better understand the evolutionary relationships of the *StHCTs* family members, multiple protein sequences of HCTs from various species were selected from the NCBI database to construct a phylogenetic tree (**Figure 5A**; **Supplementary Table S5**). The phylogenetic analysis indicates that in the HCT phylogenetic tree, the HCT protein members can be divided into 21 groups. Among them, a large number of StHCTs genes are in group 3, and many genes in group 3 have a relatively close relationship with CAT00082.1 of Coffea arabica species. Additionally, in groups 5 (StHCT7, StHCT10, StHCT15, StHCT16), 15 (StHCT6), and 20 (StHCT2, StHCT4), there are also some abundant HCT proteins. These StHCTs genes have an evolutionary relationship with HCT proteins of *Solanum lycopersicum*, *Capsicum annuum*, and *Nicotiana tabacum*, and without exception, these plants with a relatively close evolutionary relationship with StHCT are all from the *Solanaceae* family. Combined with the subsequent amino acid sequence alignment, it is hypothesized that these proteins may be related to function, and further experimental research is needed to prove this. After performing multiple sequence alignments of HCT proteins from multiple representative species (**Figure 5B**), it was found that all the aligned amino acid sequences retain the characteristic active motifs of the BAHD acyltransferase family: the catalytic center “HXXXD” and the substrate recognition “DFGWG” motif (Delporte et al., 2018). These findings also confirm the evolutionary conservation of HCT proteins in plant growth, development, and metabolism. In the inner circle of **Figure 5A**, the dicotyledonous plants (red pentagrams), monocotyledonous plants (green pentagrams), moss plants (blue pentagrams), gymnosperms (black pentagrams), and fern plants (yellow pentagrams) are respectively indicated. The outer circle indicates the plants of each familia.

**Figure 5:**
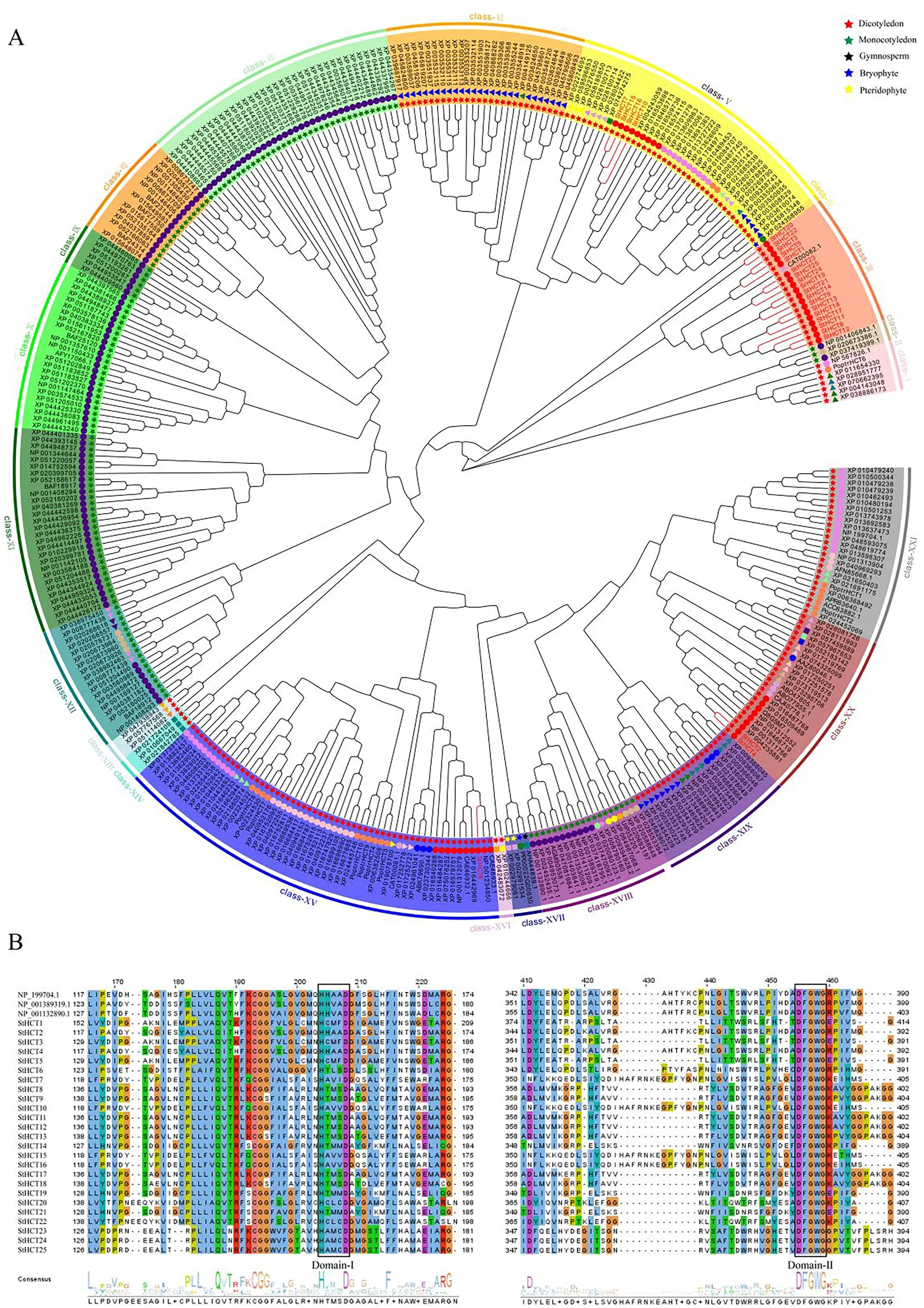
**(A, B)** The phylogenetic relationship among plant HCT proteins and the amino acid sequence alignment of some proteins with StHCTs.

### 3.6 Analysis of cis-acting elements in StHCTs genes

Through in-depth analysis of the 2,000 bp sequence upstream of the translation initiation site of the *StHCTs* genes, it was calculated and predicted that the promoters of these gene family members may contain various cis-acting elements (**Figure 6**). They are mainly divided into five types of elements: environmental response elements, hormone response elements, stage-specific elements, transcription factor binding sites, and tissue-specific elements (Environment, Phytohormone, Stage, TF, Tissue). Among them, elements involved in environmental stress and plant hormone response are particularly abundant. The promoter sequences of all genes may contain light response elements (such as numerous Box-4, G-box, GT1-motif, TCT-motif, etc.). Among the phytohormone-responsive cis-acting elements, the promoter may contain elements involved in responses to methyl jasmonate (MeJA) (TGACG-motif and CGTCA-motif), gibberellin (GARE-motif, GA-motif, TATC-box, and P-box), salicylic acid (TCA-element), abscisic acid (ABRE), and auxin (TGA-element and AuxRR-core), etc. Among the abiotic stress-responsive cis-acting elements, the promoter may contain low-temperature responsiveness (LTR) elements, MYB binding sites involved in drought-inducibility (MBS), wound-responsive elements (WUN-motif), anaerobic induction elements (ARE), and cis-acting regulatory elements involved in defense and stress responsiveness (TC-rich repeats). In addition, cis-acting elements associated with developmental stage and tissue-specific expression may include GCN4_motif, CAT-box, AACA_motif, O2-site, and RY-element. The computational prediction results described above indicate that the expression of these genes may be regulated by various signals, including light stimuli, anaerobic induction, and phytohormones, during potato growth and development. Moreover, their expression may confer stress tolerance and play important roles in defense and stress responses under adverse conditions. The left panel shows the statistical results of the predicted numbers of cis-acting elements. In the right panel, colored boxes represent different types of cis-acting elements and their predicted positions in the promoter regions of each *StHCT* gene.

**Figure 6:**
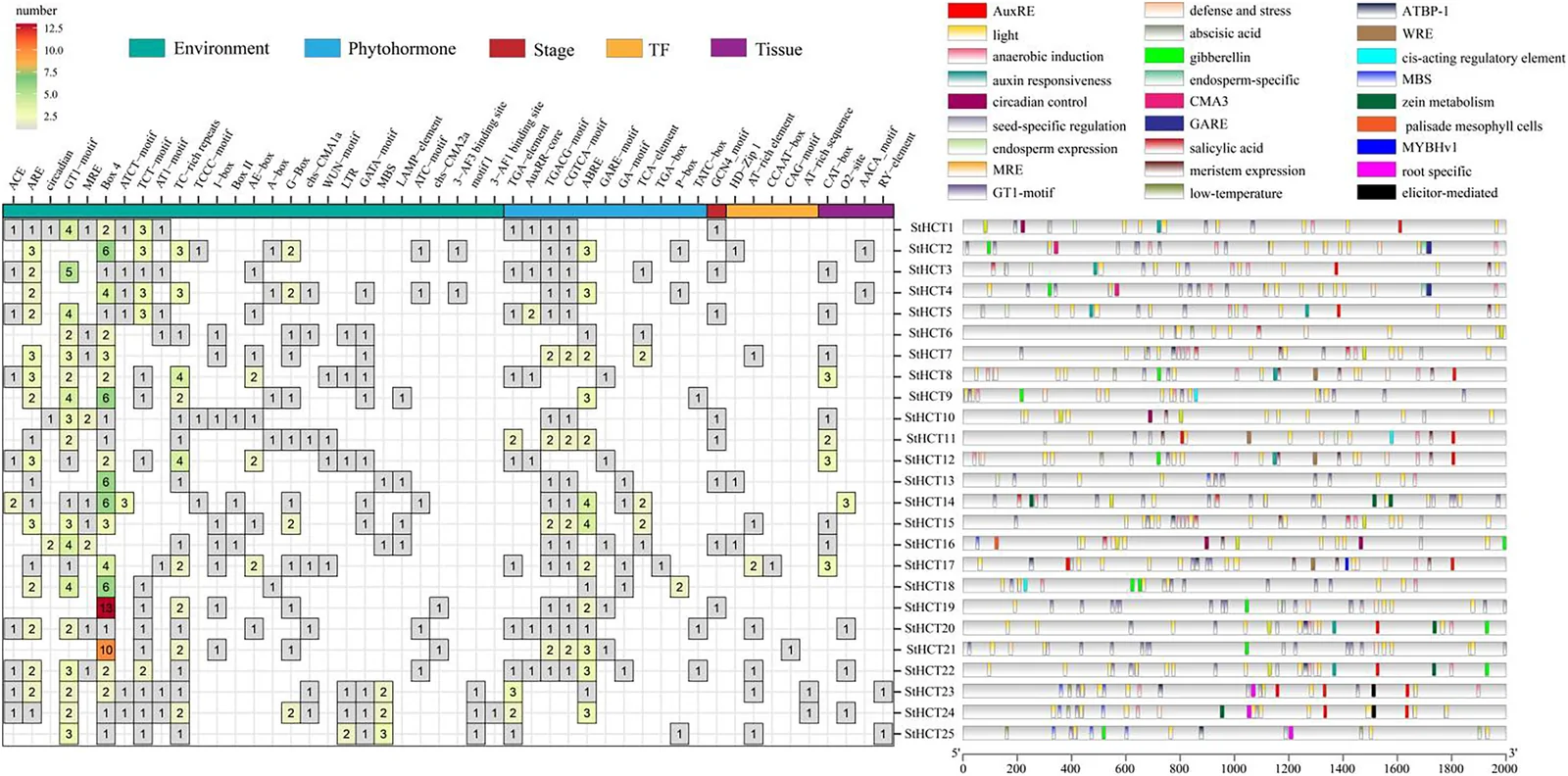
Predicted cis-elements binding sites in promoter regions of *StHCT* genes.

### 3.7 Analysis of the tissue expression patterns and stress response expression patterns of *StHCTs* genes

Using the RNA-Seq data retrieved from the PGSC database, the gene expression patterns of *StHCTs* genes in different tissues and under different stress conditions were studied (**Figure 7**; **Supplementary Table S6**). In the tissue-specific analysis, it was found that the *StHCT6*, *StHCT12*, *StHCT17*, *StHCT24*, and *StHCT25* genes had relatively high expression levels in roots, stems, leaves, and flowers, and overall, the expression of the curved prostrate stems was slightly higher than that of the expanded prostrate stems; the expression of closed flowers was higher than that of open flowers; and the expression of immature fruits was higher than that of mature fruits (except for *StHCT24* and *StHCT25* genes). Additionally, in the leaves, it was found that during the 2:00 - 22:00 period, the *StHCT14*, *StHCT19*, and *StHCT21* genes also had relatively high expression levels in the leaves, and the overall expression level changed gradually over time, showing a decreasing trend. *StHCT1*, *StHCT3* had relatively high expression levels in roots and buds, while the other *StHCTs* genes showed relatively low expression levels in the above-mentioned tissues.

**Figure 7:**
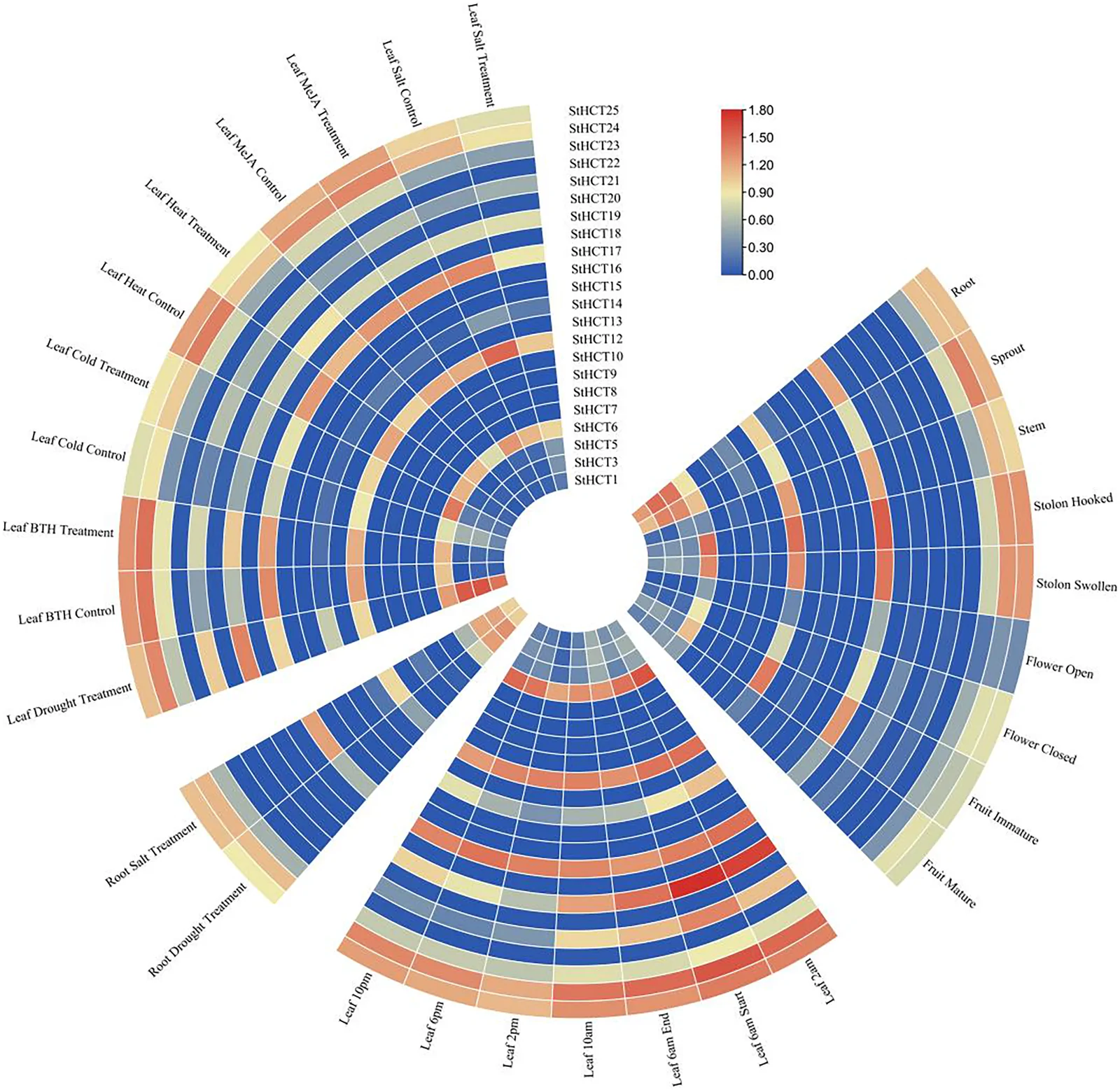
Tissue expression patterns of *StHCT* genes, as well as the expression profiles in response to adverse stresses.

After analyzing the expression profiles of these genes under different treatments, it was found that compared with other stress treatments conducted in leaves, in the root drought and salt stress treatments, the *StHCT1*, *StHCT3*, *StHCT5*, *StHCT6*, *StHCT12*, *StHCT17*, *StHCT23*, *StHCT24*, and *StHCT25* genes had obvious responses, while the expression levels of the other genes were lower. In several stress treatments in leaves, the *StHCT6*, *StHCT12*, *StHCT17*, *StHCT19*, *StHCT21*, *StHCT23*, *StHCT24*, and *StHCT25* genes had obvious higher expression levels, and these genes overall showed: the expression levels in the low temperature, high temperature, MeJA, and BTH treatment groups were higher than those in the corresponding control groups of each treatment; however, when the leaves were subjected to salt stress, the expression levels of these genes in the control group were overall higher than those in the treatment group. The study hypothesized that these genes may play important roles and have positive responses when the plants are facing adverse stress conditions. In addition, in the leaf drought treatment, *StHCT1*, *StHCT3*, *StHCT5*, and *StHCT14* also had certain expression levels. Besides, the other genes showed no response or maintained a relatively low expression level in the control group and treatment group of leaf stress treatments.

### 3.8 Interaction proteins and enrichment analysis of *StHCTs*

Protein-protein interactions (PPIs) are a crucial process in organisms, where proteins interact with each other to form complexes or multi-protein networks, thereby participating in and influencing various plant physiological activities. In this study, 10 direct interaction relationships were predicted from the proteins encoded by the *StHCT* genes (taking *StHCT6* as an example), and the interaction network is shown in **Figure 8A**. There are multiple nodes, and there are multiple sets of protein interactions between the nodes. Chalcone synthase (CHS), Esterase/lipase/thioesterase family protein, and Monoglyceride lipase (MGL) are three types of proteins that interact with each other. Among them, CHS is a key enzyme in the flavonoid biosynthesis pathway of the phenylpropanoid metabolic pathway, which is a precursor for generating numerous flavonoid compounds. Esterase/lipase/thioesterase family protein is usually involved in the biosynthesis of cutin, phloem, wax, and lipid metabolism pathways. MGL is involved in the glycerol ester metabolism pathway.

**Figure 8:**
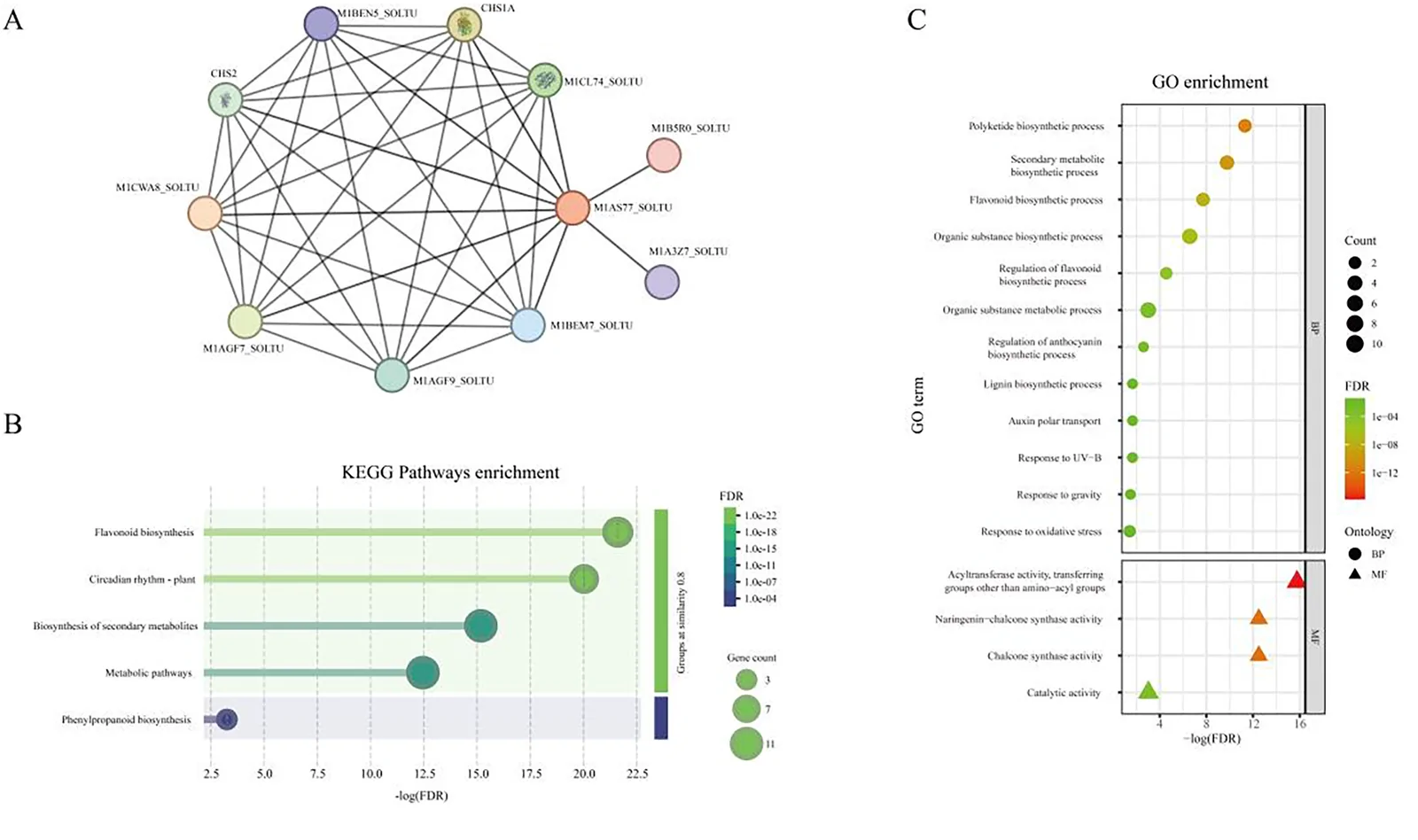
**(A–C)** PPI, KEGG and GO enrichment analysis of *StHCT* genes.

The KEGG enrichment analysis showed (**Figure 8B**) that multiple proteins interacting with *StHCT* were annotated to 5 pathways, with the main enrichment in Flavonoid biosynthesis, Circadian rhythm - plant, Biosynthesis of secondary metabolites, Metabolic pathways, and Phenylpropanoid biosynthesis. As shown in the GO enrichment analysis (**Figure 8C**), multiple proteins interacting with StHCT were respectively enriched in biological processes (Biological Process, BP) and molecular functions (Molecular Function, MF). In biological processes, these proteins mainly concentrate on Organic substance metabolic process, Organic substance biosynthetic process, Secondary metabolite biosynthetic process, etc., among which there are also processes such as Flavonoid biosynthetic process, Regulation of anthocyanin biosynthetic process, Lignin biosynthetic process, etc. In the branch of molecular function MF, these proteins mainly exert enzymatic catalytic activities, including Acyltransferase activity, Naringenin-chalcone synthase activity, Chalcone synthase activity, etc.

### 3.9 Analysis of the stress expression patterns of *StHCTs*

Gene expression is a key determinant of gene function. Based on the above analysis, some genes were selected and their relative expression levels in different stress and hormone treatments were evaluated using qRT-PCR after 24 hours (**Figure 9**; **Supplementary Table S7**). With untreated samples as the control, it was found that *StHCT6* showed an overall trend of increasing first and then decreasing in the three treatments of ABA, GA_3_, and NaCl, and its expression reached the highest value at 4 hours. In the IAA hormone treatment group, there was a certain expression level except for 12 hours; in the PEG treatment group, it maintained a continuous downward trend; in the high-temperature treatment, it had a relatively high expression level only in the first 4 hours; and in the 4°C low-temperature treatment throughout the 24 hours, it maintained a relatively low expression level. *StHCT12* showed a high expression level only in the first 4 hours of the treatment in all five treatment groups (GA_3_, PEG, NaCl, 4°C, 42°C), and its gene expression level remained at a low level during the 4h-24h period; in the ABA treatment group, the expression level showed a “up-down-up-down” pattern, with the highest expression at 4 hours; in the IAA treatment group, the expression level did not have large fluctuations and remained at a low level throughout the entire period; *StHCT17* showed a significant high expression level in the first 4 hours of the treatment in all three treatment groups (GA_3_, PEG, 42°C); and in the except for ABA treatment’s 4 hours, IAA treatment’s 8 hours, and NaCl treatment’s 2 hours, it showed a low expression level in each of the other treatment periods. StHCT19 showed a continuous decline in gene expression in all four treatment groups (ABA, GA_3_, NaCl, 42°C), with the highest expression at 2 hours of the treatment; in the IAA treatment group, it had a relatively high expression level only at 12 hours; in the PEG treatment group, it showed a “up-down-up-down” pattern; at 12 hours of the 4°C and 42°C treatment, it reached the highest expression level and then rapidly decreased. *StHCT21*, *StHCT23*, and *StHCT25* showed similar expression patterns in multiple stress treatments. Their gene expression levels reached the highest value at 4 hours in the ABA treatment group; in the GA_3_ treatment group, the expression level started to increase from 2 hours and reached the highest level at 4 hours, and then remained at a low level; in the IAA treatment group, it only had a relatively high expression level at 12 hours; in the PEG treatment group, it remained at a high level in the 4h-8h period; in the 4°C and 42°C treatment groups, it had a relatively high expression level from 2h to 4h compared to other periods, except for *StHCT23*, which maintained a low expression level throughout the treatment period in the 42°C treatment group. *StHCT24* showed certain expression levels in multiple treatment periods, generally presenting a “up-down-up-down” pattern with 1–2 cycles, and the overall expression level was close. This study hypothesizes that these genes that show prominent expression in a certain treatment and period may play an important role in the plant’s response to stress. Different letters in the figure represent significant differences between the treatment groups and the CK group.

**Figure 9:**
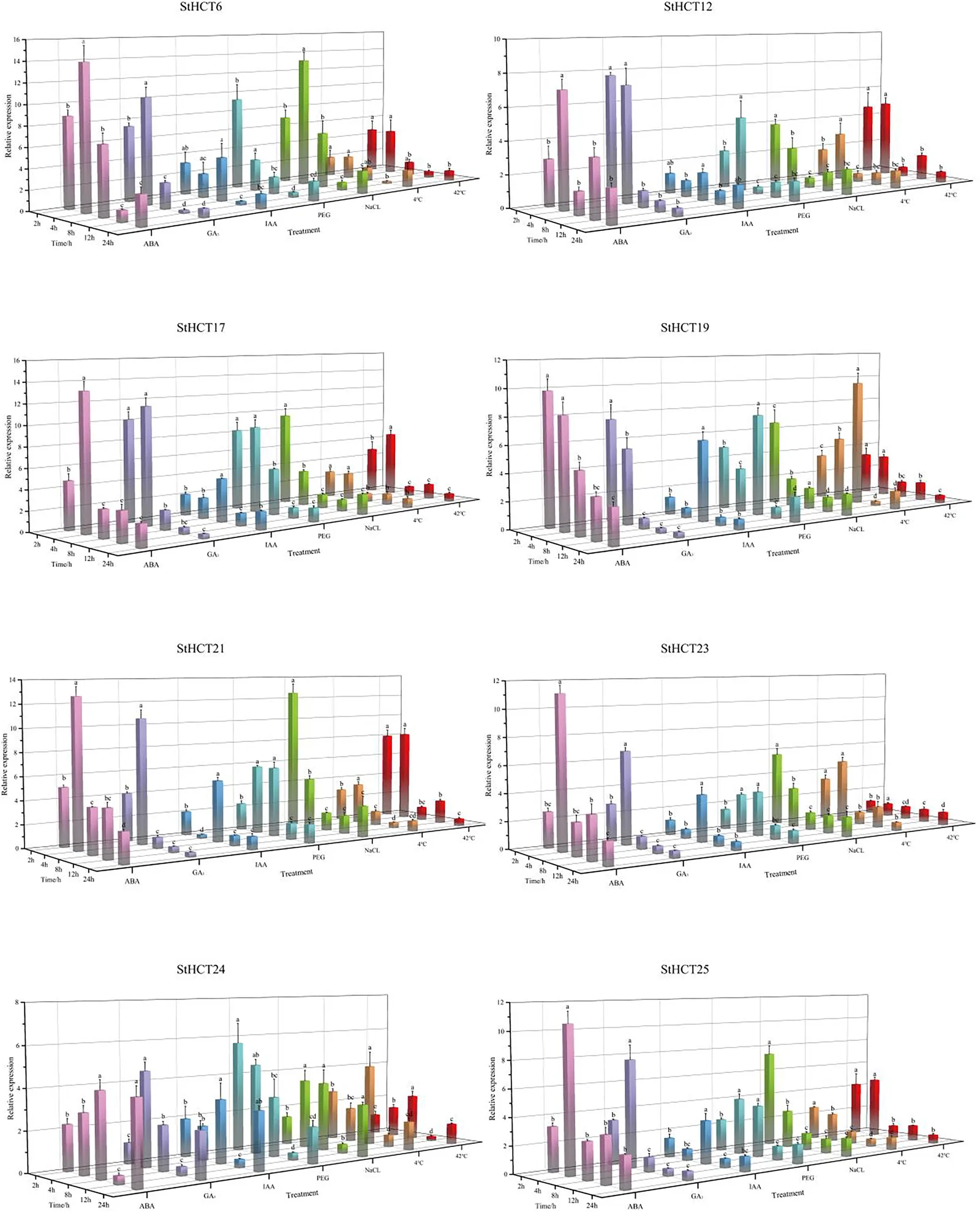
The 24-hour gene expression profiles of partial *StHCTs* in potatoes under 7 different non-biological and hormonal stress conditions.

### 3.10 Subcellular localization of *StHCT6*.

To determine the subcellular localization of *StHCT6*, a pCAMBIA2300-*StHCT6*-eGFP fusion construct was used, with the pCAMBIA2300-eGFP empty vector as the control. Agrobacterium-mediated transient transformation of tobacco leaves was performed (**Figure 10**). Before fluorescence detection, the transfected leaves were stored at 26 °C in low light for 48 hours. The results showed that the empty pCAMBIA2300-eGFP construct displayed green fluorescence signals in the nucleus, cytoplasm, and cell membrane. In contrast, the GFP fluorescence of the fusion protein pCAMBIA2300-*StHCT6*-eGFP was observed in the cytoplasm.

**Figure 10:**
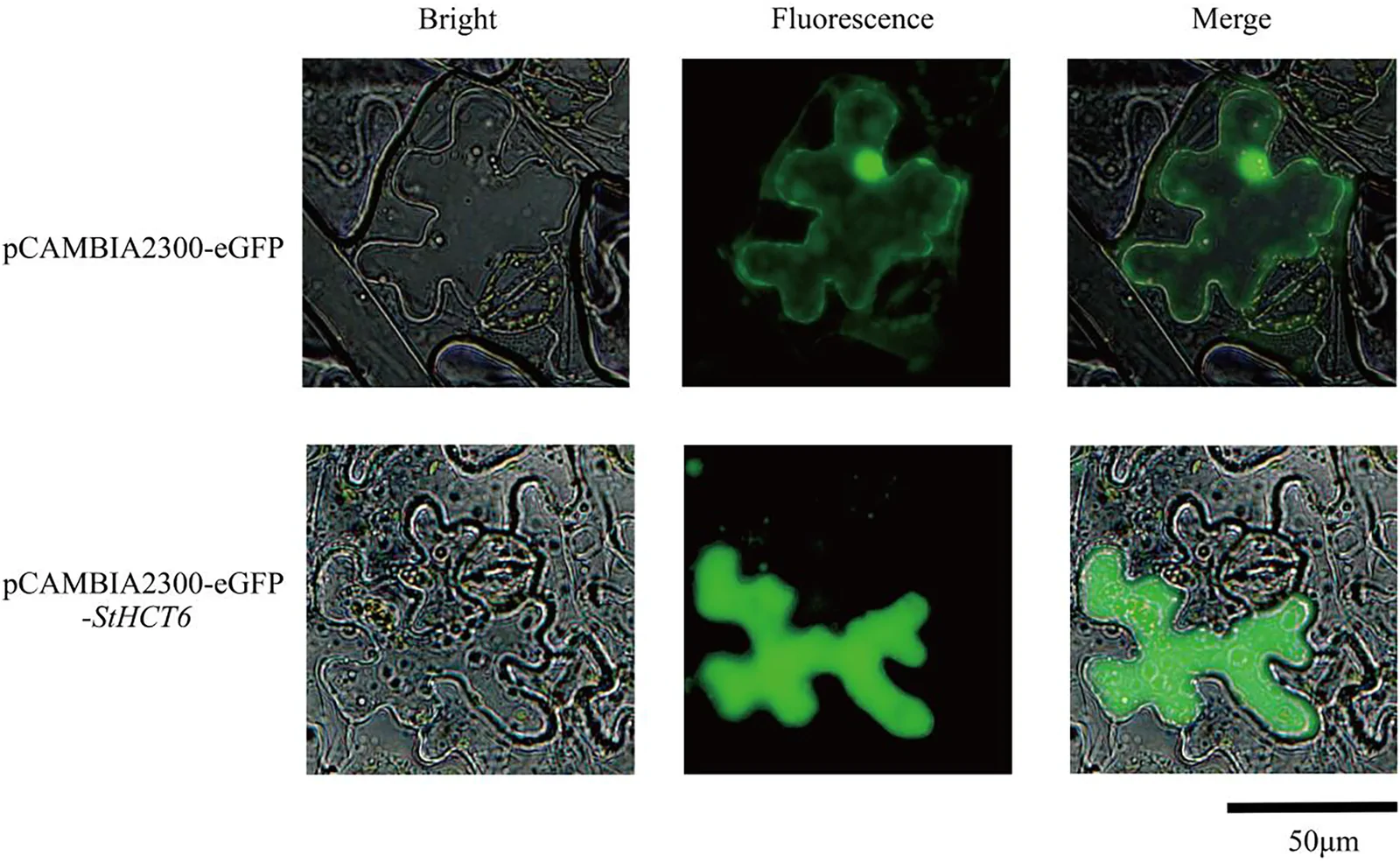
Subcellular localization of *StHCT6*.

### 3.11 Silencing of potato *StHCT6* and analysis of pTRV2-*StHCT6* phenotype and ROS damage under RS bacterial stress

Through bioinformatics analysis combined with qRT-PCR analysis, it was found that the expression patterns of the *StHCTs* family members varied under different stress conditions. After analyzing the expression patterns and conducting systematic evolutionary analysis on some *StHCTs*, it was comprehensively discovered that *StHCT6* exhibited distinct characteristics. Given that *StHCT6* encodes a key enzyme in the lignin synthesis pathway, and lignin is an important physical barrier for plants to resist pathogen invasion, this study chose bacterial infection as the stress condition for functional validation to directly assess the role of this gene in the plant defense response. The non-biological stress and hormone response observed in previous expression profile analysis further support that *StHCT6* may participate in various stress responses by regulating cell wall metabolism. To characterize the function of *StHCT6*, Agrobacterium-mediated transformation was used to achieve virus-induced gene silencing (VIGS). **Figure 11A** shows the experimental control, the left figure shows the symptomless pTRV2 empty vector plants, and the right figure shows the characteristic photo bleaching of the pTRV2-*PDS* positive control (Phytoene desaturase (PDS) as a VIGS verification marker). The silencing efficiency of pTRV2 - *StHCT6* gene was detected by qRT-PCR as 70.99%, 90.94%, 77.06%, 84.25%, and 71.23%, and the gene expression level was lower than that of pTRV2 (control) plants. Subsequently, plants with higher silencing efficiency, 2# and 4#, were selected for further experimental research (**Figure 11B**), named VIGS-2 and VIGS-4.The transcriptional profile analysis of the phenylpropanoid pathway genes (**Figure 11F**) revealed the differential regulation in the silenced plants: The expression levels of *StPAL* and *StCCoAOMT* in the WT plants were higher than those in the VIGS-4 plants but lower than those in the VIGS-2 plants; the situation for *StCOMT* was the opposite, with its expression level in the WT plants being higher than that in the VIGS-2 plants but lower than that in the VIGS-4 plants; the expression levels of *StC4H*, *St4CL*, *StC3H* and *StCCR* in the WT plants were all lower than those in the silenced plants; the expression level of *StCAD* in the WT plants was higher than that in the silenced plants. These results suggest that *StHCT6* coordinates the plant’s response to environmental changes through interacting with related genes in the phenylpropanoid biosynthesis pathway.

**Figure 11:**
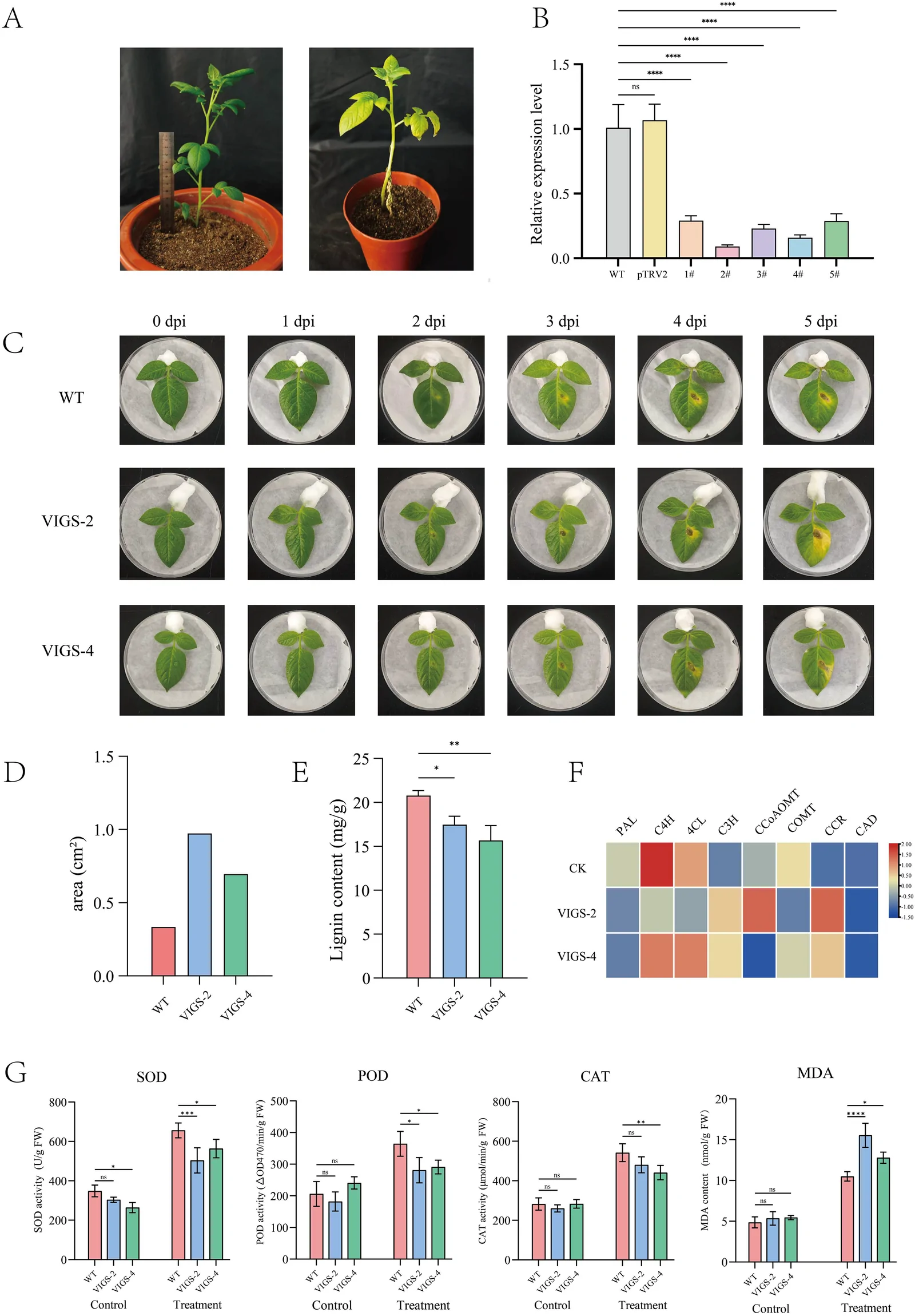
**(A–G)** The phenotype and ROS damage of pTRV2-*StHCT6* under RS bacterial stress. The experimental data are presented as the mean ± standard error of at least three independent biological replicates. The asterisks indicate the statistical significance of one-way analysis of variance: ns indicates no significant difference, * indicates p value < 0.05,** indicates p value < 0.01, *** indicates p value < 0.001, and **** indicates p value < 0.0001.

Under adverse conditions, plants produce reactive oxygen species (ROS) that induce cytotoxicity, leading to damage to plant tissue structure and programmed cell death. Comparative analysis of 30-day-old *StHCT6* silenced plants (pTRV2-*StHCT6*) and the empty vector control (pTRV2) showed differences in the response to bacterial attack under controlled light conditions. **Figure 11C** shows the progression of leaf lesions during 5-day infection, and compared to the control, the pTRV2-*StHCT6* line exhibited significantly enhanced susceptibility. The results indicated (**Figure 11D**) that the necrosis area of WT plants and VIGS-2 and VIGS-4 silenced plants increased after inoculation with RS. The necrosis area of WT plants increased by an average of 0.334 cm^2^, while that of VIGS-2 and VIGS-4 silenced plants increased by 2.08 times (0.695 cm^2^) and 2.91 times (0.973 cm^2^), respectively. The necrosis area of silenced plants was larger than that of WT plants. After conducting lignin content tests on WT and silenced plants (**Figure 11E**), it was found that the lignin content in the leaves of WT plants was higher than that of the silenced plants. These findings indicate that the silencing of *StHCT6* may affect the lignin synthesis pathway in potato leaves, thereby potentially influencing the cell wall structure in the leaves, thereby reducing the leaf’s resistance to RS infection and exacerbating the severity of leaf damage. SOD, POD, and CAT are important antioxidant enzymes in plants, and MDA is a product of membrane lipid peroxidation, whose content indirectly reflects the degree of cellular oxidative damage. To evaluate oxidative damage, the contents and activities of SOD, POD, CAT, and MDA in the leaves of *StHCT6* silenced plants and WT plants under RS stress were detected (**Figure 11F**). Before inoculation, there were no significant differences in physiological indicators of potato leaves (the SOD activity of WT plants was slightly higher than that of VIGS plants; the POD of VIGS-4 plants was slightly higher than that of VIGS plants). After vaccination, the contents and activities of SOD, POD, CAT and MDA in both WT and VIGS plants increased. Among them, the contents of SOD, POD and CAT in VIGS plants were all lower than those in WT plants, while the content of MDA in VIGS plants was higher than that in WT plants.

## 4 Discussion

We identified 25 *StHCT* genes in potato, distributed across chromosomes 3, 7, 8, and 12. This stands in clear contrast to the 53 *HCT* genes reported in Arabidopsis (Zheng et al., 2023), 82 in pear (Ma et al., 2017), and 58 in cassava (Medison et al., 2023), pointing to divergent evolutionary histories of the HCT family among plant lineages—shaped over time by ecological adaptation, historical evolutionary events, and the differentiation of duplicated homologs that can drive new functions, along with whole-genome duplication events (Zafar et al., 2022). The physicochemical properties of the StHCT proteins are broadly similar, and most are hydrophilic, a feature consistent with the involvement of hydrophilic proteins in abiotic stress responses (Zhou et al., 2018). Subcellular localization predictions placed StHCTs mainly in the cytoplasm, with a subset targeted to chloroplasts and peroxisomes, suggesting that different *StHCT* members may carry out functionally distinct roles.

Synteny analysis revealed that potato shares markedly stronger collinearity with solanaceous species such as eggplant and tomato than with other plants, a pattern of genomic similarity and divergence that sheds light on the relationships and genome evolution within the Solanaceae. The low Ka/Ks ratios observed for these gene pairs indicate that they have been subjected to strong purifying selection, retaining advantageous variants and underscoring their conservation and functional importance. Within each subfamily, the StHCT proteins carry similar conserved motifs and domain architectures; the dominant domains belong to the transferase superfamily and the PLN02481 superfamily, and motifs 1 and 4 contain the highly conserved HXXXD and DFGWG signatures, pointing to a conserved core function and potentially shared regulatory mechanisms. Members of the same subfamily also display similar exon–intron organization, and such a compact gene structure is thought to enable rapid transcriptional activation upon environmental stimuli—the fewer the introns, the faster the response, resulting in more efficient function (Vadde, 2021). Phylogenetic and amino acid alignment analyses showed that *StHCTs* possess key motifs and structural features consistent with those of model species such as *Arabidopsis*, clustering relatively close to HCTs from most other angiosperms, yet grouping more tightly with solanaceous HCTs, which hints at a direct link to their cognate protein functions. The StHCT interaction network revealed that most interacting partners participate in the phenylpropanoid pathway; notably, CHS is involved in the synthesis of precursors for numerous flavonoids. KEGG and GO enrichment analyses of these partners highlighted overrepresentation in phenylpropanoid biosynthesis, metabolic pathways, and secondary metabolite biosynthesis. Protein-protein interactions play important roles in stress responses-for instance, in maize, HCT interacts with NLR proteins to bolster disease resistance (Wang and Balint-Kurti, 2016). The promoter sequences of the *StHCT* gene family members show considerable similarity: all carry light-responsive elements and are enriched in hormone-responsive elements as well as drought-, low-temperature-, and defense/stress-responsive elements, reinforcing their involvement in stress and defense. In Arabidopsis, the transcription factor MYB15 promotes lignin deposition at pathogen entry sites by upregulating lignin biosynthetic genes including *HCT*, thereby restricting pathogen spread and potentiating effector-triggered immunity (Kim et al., 2020).

The transcriptome data revealed distinct expression patterns of these genes across different tissues. Several *StHCTs* showed higher transcript levels in more heavily lignified organs-roots, stems, and stolons-likely because they play key roles in lignin biosynthesis, whereas expression was lower in the less lignified flowers. Notably, the pronounced difference in transcript abundance between open and closed flowers warrants further investigation. Under abiotic stress treatments of leaves, some genes displayed clear responses relative to the control, a pattern broadly consistent with their expression profiles after 24 h of treatment. Upon BTH and MeJA application, these genes were expressed in both control and treated groups. Subsequent hormone treatments revealed marked responsiveness to IAA, GA_3_, and ABA.

After conducting expression pattern analysis of some genes using fluorescence quantitative methods, it was found that under high-temperature stress treatment, the expression levels of 8 *StHCTs* generally showed a high level only in the initial period of the treatment (2 h - 4 h), but in the subsequent treatment periods, their expression levels were only at very low levels. According to literature search, this study hypothesizes that when plants resist the effects of high temperature and drought, the initial stage is to accelerate transcription and translation to increase the rate of lignin synthesis in plants, improve lignification degree, thereby reducing transpiration and water loss, and reducing the harm of high temperature to the plants themselves. When analyzing the low expression levels of these genes under low-temperature stress, the speculation is that the plants may have internal mechanisms to expel water as much as possible to avoid the influence of factors such as low-temperature ice crystals on the normal growth and development of the plants. This phenomenon also occurs in *Arabidopsis* (Ji et al., 2015). For the two hypotheses of plants increasing their lignification to reduce the harm of high temperature and regulating water loss to cope with low-temperature stress, more experimental evidence is needed to verify the accuracy of this speculation, which is of crucial importance. In contrast to the early strong responses seen with ABA and GA_3_, the same genes maintained low expression throughout the IAA treatment, with only a modest increase at 12 h. A comparable situation has been reported in pepper: promoters of lignin biosynthetic genes were up-regulated by ABA and GA_3_ but showed only minor changes under SA and MeJA (Wu, 2022). Under PEG treatment, *StHCT* transcript levels rose during the early-to-middle phase and then gradually declined, whereas NaCl treatment resulted in a continuous decrease over the 24 h period. Chen et al. similarly found that most *HCT* genes were down-regulated by NaCl, PEG, and low temperature (Chen et al., 2021).

The subcellular localization results of the *StHCT6* gene indicate that the *StHCT6* protein is located in the cytoplasm, which is consistent with the localization results of the *TaHCT5B* and *ClHCT2* genes (Li, 2023; Lü, 2025). This is related to the result that the *HCT* genes catalyze the biosynthesis of phenylpropanoids in the cytoplasm. This provides assistance for further explaining the function of the StHCT protein. Lignin can provide mechanical support to plants by increasing the hardness of the cell wall and enhancing the compressive strength of the cells. In the response to pathogen infection, it can also serve as a physical defense. This study analyzed the expression levels of phenylpropanoid pathway genes in WT plants and silenced plants, and both suggested that the *StHCT6* gene may affect the plant’s response to stress. Under biotic stress, the situation of the silenced plants is the same as that of the wild plants. After pathogen infection, the contents of SOD, POD, and CAT enzymes all increased to varying degrees, indicating that the enzyme activity response of the plants after inoculation is more sensitive. By increasing the enzyme activity, the reactive oxygen species and other substances produced by the plants under stress are balanced and reduced. The difference between the two lies in that the content increase of antioxidant enzymes in the silenced plants is lower than that in the wild plants, and their antioxidant capacity is slightly weaker. Moreover, due to the relatively higher content of reactive oxygen species and other substances accumulated by the silenced plants, they may cause more serious damage to the plants. It should be noted that the isolated leaves are in an aging stress state, and their defense responses may differ from those of intact plants. Therefore, the phenotypic results of this study mainly reflect the changes in the disease resistance of the isolated leaves. This study characterized the function of *StHCT6* based on indicators such as the lesion area of the leaves, antioxidant enzyme activity, lignin content, and gene expression of the phenylpropanoid pathway. Although there may be some limitations, the evidence from multiple aspects, such as the increase in lesion area, the rise in MDA, changes in defense enzyme activity, the decrease in lignin content, and the alteration in the expression of phenylpropanoid pathway genes, mutually corroborates, preliminarily verifying that StHCT6 is involved in lignin synthesis and participates in the basic defense response. The consistent results of the two independent silenced mutant lines also to some extent enhance the reliability of the conclusion.

In conclusion, the above analysis convinces us that the research on the *HCT* gene family of potatoes has made some new discoveries and understandings compared to previous studies on other species, and has provided a certain theoretical basis for the in-depth study of gene functions in the future. This study initially explored some of the functions of *StHCT6* through virus-induced gene silencing (VIGS). However, the functions of *StHCT6* itself and those of other *StHCT* have not been deeply investigated. Moreover, the limited silencing effect conducted on isolated leaves in this study may not fully reflect the phenotype of the plant. The limitations of some experiments are still far from clarifying the path of how *StHCT* participates in the plant’s defense mechanism. In the future, we will conduct supplementary experiments or functional validations (such as overexpression and knockout) and more experimental results to verify the accuracy of this conclusion.

## 5 Conclusion

In this study, 25 members of the *StHCT* family in Atlantic potatoes were identified and analyzed. There were no significant differences in their physicochemical properties, and they showed high conservation in gene structure and evolutionary relationships. Correlation analysis indicated that this gene had a high homology with *Solanaceae* plants. Transcriptional element analysis showed that the *StHCT* gene responded significantly to abiotic stress. The analysis of the tissue specificity of the *StHCT* gene and its expression pattern under non-biotic stress using transcriptome data and qRT-PCR indicated that some *StHCTs* (*StHCT6*, *StHCT12*, *StHCT17*, *StHCT19*, *StHCT21*, *StHCT23*, *StHCT24*, *StHCT25*) had more significant responses to various stresses. The candidate *StHCT6* gene was located in the cytoplasm. After silencing *StHCT6* in potatoes, the leaves of the silenced plants suffered more severe oxidative damage after pathogen infection. These conclusions provide valuable information for further research on the biological role of *StHCT* genes in the growth process of potatoes under adverse conditions and are of great significance for potato production.

## Data Availability

The datasets presented in this study can be found in online repositories. The names of the repository/repositories and accession number(s) can be found in the article/**Supplementary Material**.
